# A different perspective on food and well-being in migrant-founded communities: seeing a Oaxacalifornia food system

**DOI:** 10.3389/fnut.2026.1771121

**Published:** 2026-04-13

**Authors:** Daniela Soleri, Violeta Jimenez, Karina Valera, Isaí Pazos

**Affiliations:** 1 Department of Geography, University of California, Santa Barbara, Santa Barbara, CA, United States; 2 Independent Researcher, Los Angeles, CA, United States; 3 Organización Regional de Oaxaca, Los Angeles, CA, United States

**Keywords:** food system, maize, Oaxacalifornia, public and planetary health, traditional foods

## Abstract

Our food system is harming individual, public and planetary health, and scientists and practitioners have called for a “Great food transformation…to normalize healthy diets from sustainable food systems.” One reason this transformation is needed is due to a series of health-impacting transitions in diet and health resulting from lifestyle and food changes. Migrants encountering a new food environment and the subsequent dietary, nutritional, and epidemiological transitions are at increased risk of acquiring noncommunicable diseases. The challenges to health and well-being in migrant-founded communities are real; however, we consider how a different perspective could offer alternative pathways for achieving food transformation and strengthening well-being. These communities are typically depicted as experiencing a dietary transition due to being passive, limited-resource, limited-ability consumers in a novel, health-compromising food environment. But some migrant-founded communities selectively resist such transitions including through culture and identity expressed in traditional foods. We argue for this contrasting perspective regarding traditional maize-based cuisine in Oaxacalifornia, specifically the Oaxacan heritage community in Los Angeles, California. We outline some of the implications of this perspective for community well-being, research and practice.

## 1 Introduction

The global food system, from production [e.g., (1)] to consumption (2) and waste, is harming individual, public, and planetary health (IPPH) (3). This food system is characterized by a shift toward more uniform repertoires of food crops and diets within and across nations (4), and from more nutrient-dense, plant-rich traditional diets to less healthy ones containing highly processed, energy-rich, nutrient-poor foods and more animal products. Not all traditional foods are healthy, but dietary recommendations can undermine well-being if they lack an understanding of traditional foods. For example, for the Zapotec population in the Central Valleys of the southern Mexican state of Oaxaca, a maize and cacao-based beverage—tejate—is an important element in rural households’ diets and cultural identity (5). However, public health workers sometimes advise against drinking tejate because it is often sugar-sweetened, increasing the risk of dysglycemia (6). Yet the most common substitute for tejate is sugar-sweetened soda with a much higher glycemic index, lower satiety, and none of the nutritional and sociocultural benefits of tejate.

The dietary change away from traditionally based foods and a subsequent epidemiologic shift toward higher mortality from non-communicable diseases (NCDs) such as type 2 diabetes (T2D), comprises the ongoing “nutrition transition” (7, 8), which impacts lower resource households and communities disproportionately. This has been recognized for decades, e.g., the “commerciogenic malnutrition” resulting from corporate pressure for infant formula (9), negative dietary changes from the “coca-colonization” observed in Yucatan, MX (10). Still, implementing participatory, equitable responses remains a challenge.

To stop and reverse multiple harms scientists have called for a “Great food transformation…[an] unprecedented range of actions taken by all food system sectors across all levels that aim to normalize healthy diets from sustainable food systems,” and recommended a Mediterranean-style planetary health diet (PHD) (11). In response to criticism (12), the PHD has recently been broadened to include healthy traditional diets [e.g., (13, 14)].

## 2 The traditional Mexican food system’s relevance for the nutrition transition

What is today southern Mexico is the center of origin, domestication and diversity of maize (*Zea mays* L). In the approximately 9,000 years since they began domesticating maize there (15–17), indigenous people have developed a complex, nutritionally complete, regionally diverse cuisine built on the milpa farming system, a diverse polyculture based on maize, with other crops and wild plants.

Milpa and similar biodiverse, small-scale production systems are increasingly recognized as important alternatives to industrial production, with more positive outcomes for climate, environment and social justice (18, 19), and evolutionary processes supporting agricultural sustainability (20).

However, Mexico’s indigenous agriculture, agrobiodiversity and cuisine have been eroded by globalization and trinational neoliberal agreements (21, 22). Multinational corporations aggressively promoting and selling processed foods have saturated the country (23) with large negative health impacts, especially in indigenous communities (24–26). In 2018 about 80% of new T2D cases in Mexico were diet-related (27).

In response, Mexico has taken steps to recognize and build on its long history of traditional crops and diets, defining a culturally salient diet with benefits for IPPH based on its rich agrobiodiversity and culinary traditions (28). Traditional Mexican cuisine is globally acknowledged as an “Intangible Cultural Heritage of Humanity” (29), and in March 2025 Mexico amended its constitution, declaring native maize an “element of national identity,” critical to food sovereignty, and a biocultural heritage meriting protection from biotechnologies (30).

## 3 Migration, food, and health

The nutrition transition is observed in populations worldwide and can be especially rapid among migrants (31) acculturating to the new food environments they encounter (32, 33). The food environment “contains the total scope of options within which consumers make decisions about which foods to acquire and consume” (34) and is a focus of research and practice related to migrant food experiences.

The food environments of low-income communities, including migrants, in the US are dominated by lower-cost commodity-based foods. US data for 2001–2014 showed poorer households’ diets contained a greater proportion of commodity-based foods, and consumption of commodity-based foods was positively correlated with higher obesity and T2D prevalence (35). That is, the food environments most available to migrants and other low-income communities in the US are obesogenic and dysglycemic.

Understandably, public health and community organizers frequently assume that migrants are experiencing a nutrition transition as passive, limited-resource, limited-knowledge consumers operating in a novel, health-compromising food environment.

In new food environments migrants must negotiate new foods, limited time and resources, and pressure from their more rapidly acculturating children, all contributing to dietary change (36). Still, migrants often recreate their traditional foods, sometimes through “migrant marketplaces…transnational urban centers constituted by physical and imagined linkages between mobile people and the traveling foods and culinary experiences that follow them” (37). While many traditional foods, or current versions of them, are positive for IPPH, others can be adjusted to be healthier than industrial foods (e.g., (28, 38)), and these foods also provide identity, sociocultural connection and enjoyment, helping avoid the “cultural bereavement” that may occur with migration (39).

## 4 Oaxacalifornia

The state of Oaxaca has the greatest biological and cultural diversity in Mexico (40). In 2020, over 29% of its population of 4.13 M spoke an indigenous language, and over 20% of employed people worked in agriculture (41).

Migration is a survival strategy that requires indigenous migrants navigate discrimination and marginalization, including in Mexico (42). Stephen uses the term “transborder” to emphasize that indigenous Oaxacan migrants cross multiple borders, “ethnic, cultural, colonial and state borders within Mexico” (43), and for some, the US-Mexico border. For indigenous Oaxacan migrants to the US, maintaining ties with their sending community [e.g., see (44)] helps them meet the challenges of discrimination and marginalization.

Indigenous Oaxacans have engaged in labor migration for generations within Mexico and beginning in the late 20th century to the US, including California, reflecting political and economic conditions in both countries. For example, after NAFTA went into effect in 1994, dumping of subsidized US maize eliminated 58% (~4.9 M) of family farm jobs in Mexico, resulting in a major wave of outmigration (45, 46).

Los Angeles (LA) has been a center of Oaxacan migration for decades; population estimates of Oaxacans in LA are of poor quality, but the number is large. One estimate of Zapotec-heritage Angelinos was 200,000 in the early 2000s (47). Oaxacalifornia as a transborder entity was defined by Kearney as “a third sociocultural and political space” (48), clarifying that Oaxacalifornia is “a popular term…of unknown origin” (49). Rivera-Salgado (50) describes how the close relationship between migrants in California and their place of origin in Oaxaca, centered on indigenous identity, has created one continuous community, Oaxacalifornia. We define Oaxacalifornia as a communal construction of indigenous Oaxacans of an alternative space encompassing both Oaxaca and California, and the networks, processes and relations that maintain it. In LA, the Oaxacalifornia community is diverse and spatially stratified by ethnic group (CIELO)^1^ with downtown and West LA dominated by Zapotec-speakers, especially from the Central Valleys and the Sierra Norte of Oaxaca. These are the people we have been listening to.

## 5 Why do we see a food system?

**Figure 1:**
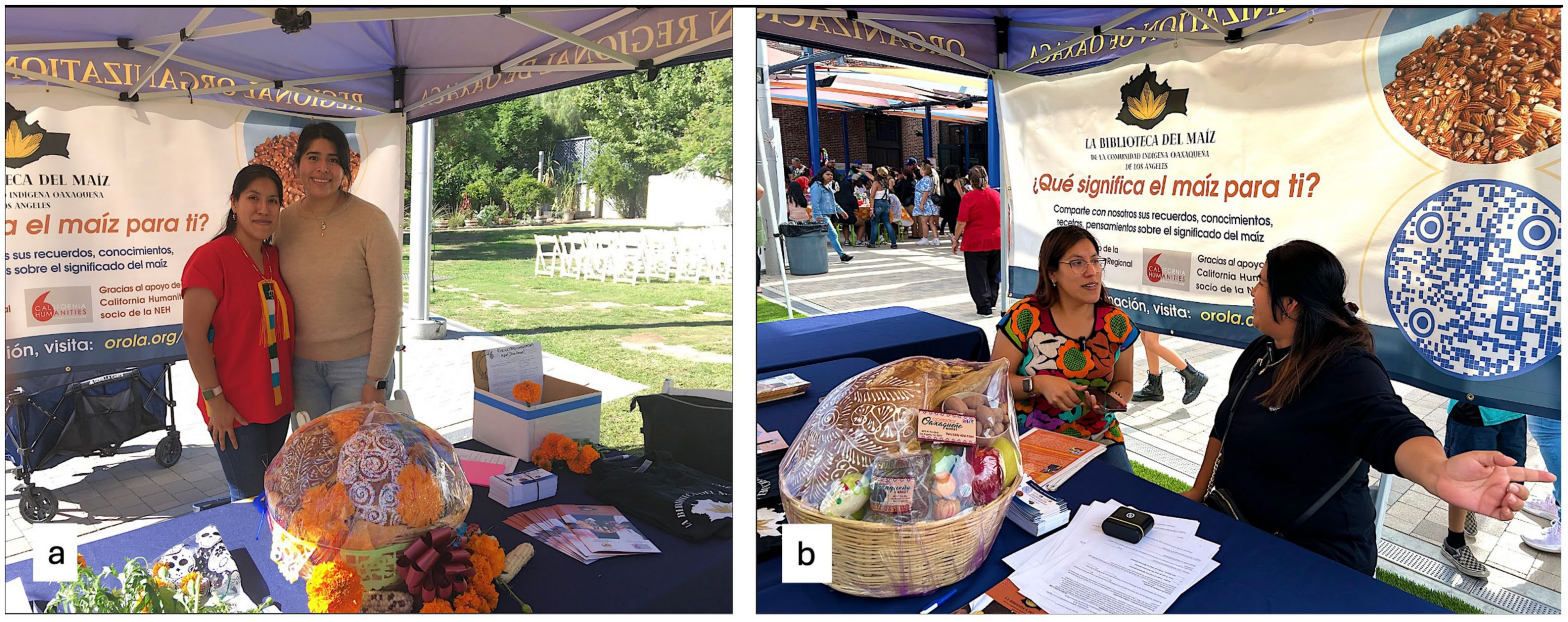
The Biblioteca del Maíz table at ORO-organized community events in **(a)** 2023 with coauthors KV and VJ, and **(b)** 2024 with KV interviewing a community member.

For the last 2 years we have worked together on the Biblioteca del Maíz (BdM)^2^, a small project of the Organización Regional de Oaxaca (ORO)^3^, a 38 year old cultural nonprofit founded and run by Oaxacan migrants in LA. The BdM solicits public statements about the meaning of maize, and traditional maize foods at public events organized by ORO for LA’s Oaxacalifornia community (Figure 1). We also interview community experts who have played a significant role in bringing Oaxacan foods to Oaxacalifornians in LA (Soleri et al. ms in preparation).

**Figure 2:**
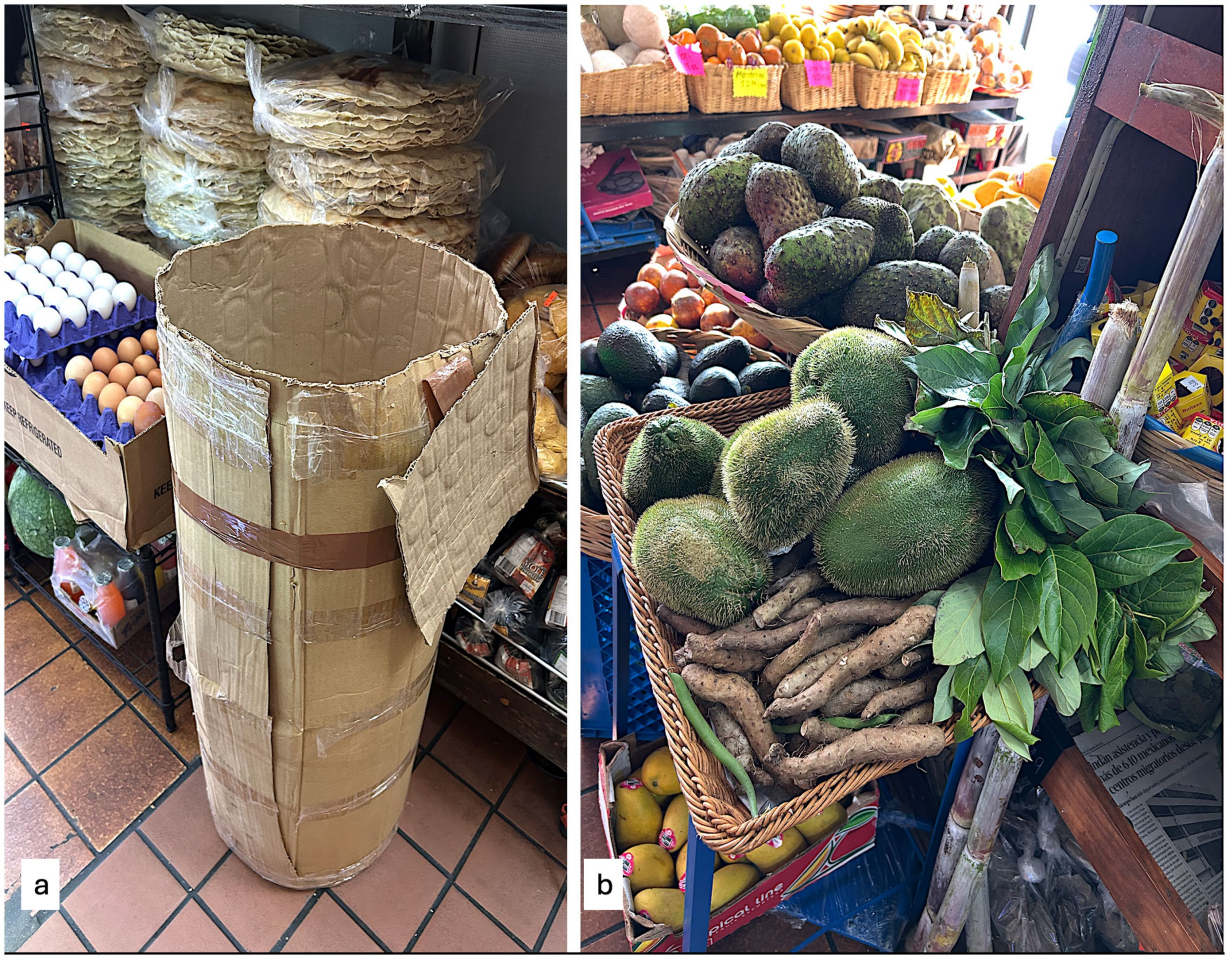
Traditional foods and ingredients from Oaxaca made available in LA, CA; **(a)** tlayudas and shipping package, **(b)** produce including chayote (*Sechium edule*) and avocado (*Persea americana*) leaves.

We are learning how Oaxacalifornians have changed their food environment in ways we think are best described as building parts of a food system. Like Oaxacalifornia itself, these changes are based on close links between people in LA, their families and natal communities. Envíos (international shipments of small care packages) from family in Oaxaca were initially the only sources of Oaxacan specialties such as tlayudas (handmade, dry maize tortillas, ~30 cm diameter), chocolate, chapulines (seasoned, toasted grasshoppers), and mole [e.g., (44), also (51)]. Starting in the late 1980s, ingredients or prepared foods were sold from apartments, the back of trucks, or by street vendors as the LA community grew and could support more food imports. The mid 1990s saw the establishment of Oaxacan restaurants and small stores selling ingredients and foods brought from Oaxaca (52, 53). Pioneering restaurateurs and shopkeepers describe how they used direct family and hometown connections with farmers, gardeners and food artisans to offer products and prepare dishes sought by fellow Oaxacalifornians, the vast majority of their customers (Figure 2). To do this they created transport networks, identified or developed appropriate import protocols, including health inspections, for foods brought to LA from Oaxaca. Oaxacalifornian food artisans, entrepreneurs, and consumers are creating a food system built on personal transnational ties and deep cultural meaning. The robust demand in LA for some traditional foods and how they are produced undoubtedly affects IPPH across Oaxacalifornia.

Tlayudas illustrate the strong interconnections across Oaxacalifornia that form a food system. The traditional size, moisture content, and flavor of tlayudas are highly preferred by discerning customers of Oaxacan heritage, making them a staple that business owners import directly from their communities, with demand growing substantially since the early 1990s. Some studies suggest that this increasing demand for artisanal tlayuda production and export have increased the number and incomes of tlayuderas (54, 55), but has also pushed them to procure non-local maize for their production (56).

Another example illustrates the direct health benefits of recognizing a Oaxacalifornia food system. Elevated blood lead levels in a migrant population in Seaside, California were traced to the glaze on ceramics used to prepare chapulines in Oaxaca (57), which were then brought to Seaside in envíos. Despite a lack of government and public health action, researchers and community groups in Oaxaca and California developed educational programming to alert affected communities.

## 6 Discussion

We believe there are large potential benefits in recognizing a Oaxacalifornia food system, especially if we pay attention to nuance [see (58)].

*First*, recognition would shift the discussion of individual and public health from one defined by deficits - financial, experiential, informational, to one defined by assets - historical, sociocultural, agricultural, culinary, and institutional.

*Second*, an asset-based perspective opens the door to genuine partnerships with community experts, could support equity and justice, and for that reason has been promoted for more effective public health work (59–61). An asset-based perspective acknowledges injustice, bias and harm, but unlike deficit or “damage-centered” approaches (62), initiates change through the aspirations and assets present in the community itself.

*Third*, this recognition would help move the discussion of individual, public and planetary health beyond narrow disciplinary and professional boundaries, encouraging a more holistic approach and sharing of resources and methods developed across Oaxacalifornia.

*Fourth*, recognition would acknowledge the reality of transborder processes so that these can be better understood through research and policy for the benefit Oaxacalifornians, and the greater public good, including IPPH.

Seeing a Oaxacalifornia food system can shift thinking of traditional diets as nostalgic, to their relevance for providing IPPH benefits. It also recognizes the expertise and effort that created it, and its biocultural significance. Now is an opportune time for this recognition, as younger Oaxacalifornians are taking new pride in their heritage including foods, and US federal actions against all migrants have become egregiously cruel. The first step is for us, as researchers and practitioners, to see that food system.

## Data Availability

Publicly available datasets were analyzed in this study. This data can be found at: http://www.orola.org/maiz.html. Given the current federal intimidation of migrant communities in the US, some of the data have been withdrawn from the public webpage listed. Queries can be directed to the corresponding author.

## Footnotes

^1^ https://storymaps.arcgis.com/stories/618560a29f2a402faa2f5dd9ded0cc65/

^2^ http://www.orola.org/maiz.html

^3^ http://www.orola.org/index.html
